# Fibrinolytic Therapy in Purulent Pericarditis

**DOI:** 10.31083/j.rcm2401017

**Published:** 2023-01-10

**Authors:** Małgorzata Dybowska, Monika Szturmowicz, Katarzyna Lewandowska, Małgorzata Sobiecka, Witold Tomkowski

**Affiliations:** ^1^1st Department of Lung Diseases, National Tuberculosis and Lung Diseases Research Institute, 01-138 Warsaw, Poland

**Keywords:** purulent pericarditis, pericarditis, intra-pericardial fibrinolysis

## Abstract

Purulent pericarditis (PP) is rare disease, and if left untreated, it is associated with very high mortality, nearly 100%. A considerable clinical problem due to PP is a very high probability of developing constrictive pericarditis (CP). Pericardial drainage is essential in the treatment of PP and should be performed urgently. The use of broad-spectrum antibiotic therapy is equally important. Unfortunately, fibrin deposits often create occulated spaces and reservoirs that reduce the penetration of antibiotics and their effectiveness. The rationale for the intrapericardial use of fibrinolytic drugs in PP is based on their ability to dissolve fibrin strands and collagen fibres, thus improving the penetration of antibiotics to the pericardial sac and lowering the risk of CP. The choice of the drug, as well as its dosage and the method of administration is still under debate. The authors of the article share their experiences and review current literature on this rare topic.

## 1. Introduction

Purulent pericarditis (PP) is defined as an infection localized in the pericardial sac, that results in the production of macroscopically or microscopically purulent fluid [1].

PP is considered to account for only 1% of all pericardial diseases [2]. It may be primary or secondary to another infectious process. Both types of PP are very rare, and primary ones are extremely rare, especially in European countries [1, 2, 3]. The authors of the article, who have been working for 36 years in the centre dealing with pericardial diseases, have encountered only a dozen cases of PP.

If left untreated, PP is associated with nearly 100% mortality [4]. Overall mortality of patients with treated PP is 10–15%. Most challenging clinical problem is a very high probability of the development of constrictive pericarditis (CP), which may occur very early after the onset of PP [2, 5, 6].

The risk of developing pericardial constriction in the patients diagnosed with PP is classified as high, and reaches about 20–30%, accounting for approximately 3–6% of all causes of CP [2].

## 2. PP Pathogenesis

PP may be a complication of an ongoing inflammatory process localized in the mediastinum, pleura, palatine tonsils, or - in the chest wall.

It can also develop in patients with congenital or iatrogenic immune disorders for example, during immunosuppressive therapy or the use of chemotherapy.

There are five pathomechanisms of infection in secondary PP:

(1) contiguous spread from an intrathoracic site;

(2) hematogenous spread;

(3) extension from a myocardial site;

(4) perforating injury or surgery;

(5) extension from a subdiaphragmatic site [7].

In developed countries the most common microorganisms, responsible for PP development were staphylococci, streptococci and pneumococci, while the dominant accompanying lesions were empyema (50%) or pneumonia (33%) [2, 5].

Staphylococcus aureus and fungi are more common pathogens in immunocompromised patients or after thoracic surgery [2, 7].

## 3. PP Symptoms 

PP is usually an acute fulminant illness. Most often it develops suddenly, with a high fever. In addition, shaking chills, night sweats and dyspnoea are common. In most patients typical pericardial chest pain is absent. In many cases, the pericarditis remains unsuspected because of dominant presence of symptoms and signs related to an underlying infection, such as pneumonia or mediastinitis, following complicated thoracic surgery or trauma.

## 4. PP Diagnostics

**Fig. 1.**
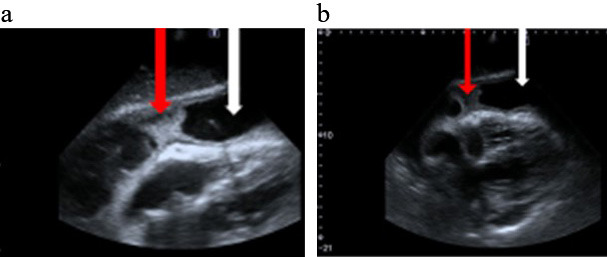
**Bedside echocardiography (a,b) Modified sub-sternal views**. White arrow a large amount of fluid in the pericardium. Red arrow fibrin deposits in the pericardium space.

**Fig. 2.**
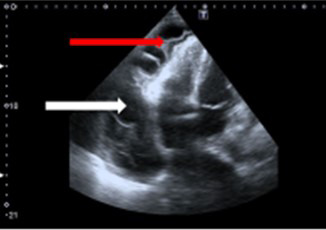
**Bedside echocardiography**. A modified apical view. White arrow a large amount of fluid in the pericardium. Red arrow fibrin deposits in the pericardium space.

In the initial phase of PP the diagnosis may be facilitated by an echocardiographic image suggesting a purulent aetiology of the pericardial fluid: a dense fluid character with the presence of an epicardial fibre layer or the formation of a fluid space separated by fibrin deposits (Figs. 1,2).

Routine electrocardiography (ECG) may suggest pericarditis, though there are no specific changes is ECG in PP. Abnormalities depend on the amount of fluid in the pericardium or the development of CP [3]. ECG may demonstrate non-specific ST-T wave changes, diminished QRS and T-wave voltages, PR-segment depression, bundle branch block, and electrical alternans of QRS, rarely T waves (but this is rarely seen in the absence of tamponade) [3].

**Fig. 3.**
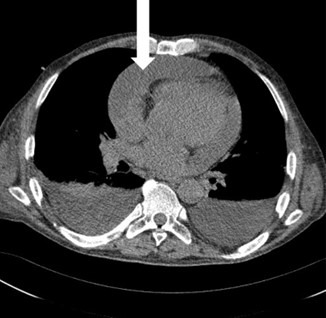
**Chest CT scan**. White arrow a large amount of fluid in the pericardium.

Valuable information on the character of the pericardial fluid is pded by the chest computed tomography (CT) image with the assessment of the attenuation value of the fluid (Hounsfield units–HU). Values between 20–60 HU suggest a purulent aetiology [8] (Fig. 3).

Suspicion of the presence of pus in the pericardial sac is an indication for the exploration of the pericardial space.

## 5. Treatment

Pericardial drainage is crucial and should be performed urgently in PP.

The fluid may be purulent or turbid, with high white blood cell counts (>10,000/μL) and high levels of neutrophils in the smear [2, 3]. Characteristic of the bacterial aetiology is also the low level of glucose in pericardial fluid and a low pericardial fluid/serum glucose ratio (mean 0.3) [2].

Pericardial fluid cultures should be taken for bacteria, mycobacteria (tuberculosis and non-tuberculous mycobacteria) as well as fungi. Cultures of blood or other materials may be performed, as guided by the clinical presentation [2].

It is very important to apply broad spectrum antibiotics, as quickly as possible, and once the pathogen has been established, the therapy directed by the sensitivity of the bacteria, should be provided.

Pericardial fluid drainage and the use of antibiotics, that penetrate well into the pericardial sac, are usually not sufficient for effective therapy. The occurrence of occulated, purulent effusion, separated by fibrin bridges, limits the effectiveness of drainage and antibiotics.

Pericardiotomy (creating a pericardial window via sub-xiphoid route) is the preferred method of surgical treatment recommended by the European Society of Cardiology Guidelines, as it is associated with a highest effectiveness of drainage and lowest incidence of CP [2].

CP is a rare but serious consequence of PP characterized by a loss of pericardial elasticity, which leads to a reduction in the proper filling of the heart chambers during the diastolic phase. If untreated—it is a progressive disease with a poor prognosis [2, 3].

Rationale for the use of fibrinolytic drugs in PP is based on their ability to dissolve fibrin strands and collagen fibres, occluding pericardial cavity. The protease fibrinolytic drugs (streptokinase (SK), urokinase) as well as recombinant tissue plasminogen activator (r-tPA), convert inactive plasminogen into the active lytic enzyme-plasmin.

Thus, the goals of direct administration of fibrinolytic therapy into the pericardial sac are the following:

(1) release of purulent pericardial exudate loculated in adhesions;

(2) dissolving the accumulated collagen fibres (the layer surrounding the pericardium, which accumulates bacterial films);

(3) relieving the pericardium from connective tissue adhesions, which reduces the likelihood of developing CP;

(4) facilitating the penetration of antibiotics, and thus increasing the effectiveness of antibacterial treatment.

There is a very limited experience with the use of fibrinolytic drugs in PP. So far, there was only one randomized trial on this issue published by Cui *et al*. [9] in 2005. This study investigated the efficacy of intrapericardial fibrinolysis with urokinase in preventing CP, in patients with infective pericardial effusions. A total of 94 patients diagnosed as infectious exudative pericarditis (34 patients with PP and 60 with tuberculous pericarditis, the disease duration was less than 1 month in all the patients). Patients were consecutively recruited from 1993 to 2002. All individuals were randomly given either intrapericardial urokinase along with conventional treatment in study group, or conventional treatment alone (including pericardiocentesis and drainage) in control group. The dosage of urokinase ranged from 200,000 to 600,000 U (mean 320,000 ± 70,000 U). Intrapericardial bleeding related to fibrinolysis was observed in 6 of 47 patients. The duration of follow-up ranged from 8 to 120 months (mean 56.8 ± 29.0 months). During follow-up, there was no cardiac death, and pericardial constriction events were observed in 9 (19.1%) of study group and 27 (57.4%) of control group. The authors found that the early use of fibrinolysis was a safe intervention, that enabled the complete evacuation of pericardial effusion and significant reduction of the risk of pericardial constriction [9].

As PP is recognized rarely, most data concerning the efficacy and safety of intrapericardial treatment come from case reports or case series.

The authors’ experience concerning intra-pericardial treatment includes the use of SK and—in recent years—the use of r-tPA [10, 11, 12, 13]. Intrapericardial fibrinolysis was applied in 7 patients with PP treated in our centre (in 3 patients r-tPA was applied; 2 patients needed repeated intrapericardial dose of r-tPA and too in 3 patients SK was given and one patient was treated by both fibrinolytics) [10, 11, 12, 13].

The purulent pericardial fluid was obtained from all of the patients, the cultures of the fluid tested positive for methicillin-sensitive Staphylococcus aureus (MSSA) in one patient, Streptococcus species in the second; Streptococcus intermedius in the third and Staphylococcus epidermidis in another (Staphylococcus epidermidis was diagnosed in two different fluid cultures thats way it was considered a cause of PP, not a contamination). In the other patients, the cultures of pericardial fluid were negative, probably due to previous antibiotic therapy. Testing for tuberculosis (bacterioscopy and pericardial fluid culture) were negative.

Broad-spectrum antibiotic therapy was used intravenously in all cases, on average for 21 days.

Subxiphoid pericardiotomy under general anaesthesia has been performed in all of the patients with implantation of a large tube drain (Pezzer drain). The indications for intrapericardial fibrinolytic treatment used in the authors’ centre, were: prolonged purulent drainage and fibrin deposits in pericardium, early echocardiographic signs of pericardial constriction despite optimal treatment, excessive pericardial drainage with signs of pericardial fluid inoculation [10, 11, 12, 13].

SK or r-tPA were applied by a large pericardial drain (Pezzer drain). The tube was closed and re-opened after 12 h (SK) or 24 h (r-tPA).

SK was applied at a dose of 500,000 IU dissolved in 50 mL normal saline and r-tPA was applied at a dose of 20 mg dissolved in 100 mL of normal saline. Length of drainage ranged 17 to 32 days.

Fibrinolytic treatment was effective in all of the patients, reducing the echocardiographic signs of early constriction and reducing large pericardial drainage [10, 11, 12, 13].

No serious complications, such as bleeding, allergy or hypotension, were noted [10, 11, 12, 13]. In one case treated by r-tPA, extensive leak of pericardial fluid next to the drain was observed and it was reopened after 5 h [13].

Augustin *et al*. [14] in 2011 and Wiyeh *et al*. [15] in 2018 summarized clinical experience with intrapericardial fibrinolysis and both concluded that it may effectively prevent the development of CP. Majority of the patients have been treated with intrapericardial SK. Several non-fatal complications have been reported: one case of cardiac tamponade due to bleeding, a few cases of irrelevant bleeding into the pericardial sac and, occasionally, hypotension, fever and fistula formation [14, 15].

Effectiveness of intrapericardial fibrinolysis has not been analysed in prospective clinical trials so far.

In the authors’ clinical practice, fibrinolytic drugs were administered in cases of prolonged drainage of purulent pericardial fluid, persistent large volumes of purulent pericardial drainage, or when echocardiography revealed fibrin deposits inside the pericardium or on the epicardium [10, 11, 12, 13]. According to the European Society of Cardiology (ESC) Guidelines 2015, echocardiography is the basic and readily available diagnostic method in the diagnosis of constrictive changes in the pericardium, especially in their initial stage and can be repeated every day during the treatment with fibrinolytic agents [2]. The ineffectiveness of drainage and/or the antibiotic treatment prompted the use of fibrinolytic drugs [10, 11, 12, 13].

In the observed cases, intrapericardial fibrinolytic therapy was a turning point in the treatment efficacy [10, 11, 12, 13].

Therefore, the question arises whether, at the time of diagnosis of PP, the use of intrapericaridal fibrinolytic drugs would not be justified. This raises the question of the rationale of early intra-pericardial fibrinolysis, immediately after the diagnosis of PP is made. Nevertheless, the effectiveness of such policy requires further confirmation in clinical trials.

Future goals for the research community are:

(1) evaluation of the efficacy and safety of fibrinolytic drugs applied directly into the pericardial space;

(2) determining the most effective and safe drug and its dose;

(3) determining the optimal timing of the procedure;

(4) answering the question, whether repeated administration of fibrinolytic drugs to the pericardial sac may increase the effectiveness of treatment, without compromising its safety.

SK, which is a product derived from the Streptococcus heamolyticus, can cause severe allergic symptoms, especially when repeatedly administered. For this reason, its repeated use in patients with PP should not be recommended.

Because of the risk of allergy and immunization, urokinase or r-tPA use has been suggested for repeated administrations [16].

The formation of collagen fibres in the purulent pericardial exudate is a defence mechanism of the organism and serves to limit the spread of infection by trying to limit it in closed spaces. On the other hand, their presence hampers the penetration of antibiotics and reduces their effectiveness.

The effect of administering fibrinolytic drugs directly to the pericardial sac, is a high concentration of plasmin, which dissolves collagen fibres (not fibrin, which is the final product of the activation of the coagulation processes). Moreover, the dissolution of connective tissue bridges causes easier penetration of antibiotics, which facilitates the inhibition of the inflammatory process. Reducing the time of inflammation in the pericardial space may additionally improve the elasticity of the pericardial sac. It is likely that these mechanisms are responsible for reducing the risk of developing CP. In cases treated at the authors’ centre the influence of intrapericardial fibrinolysis on the coagulation system was checked, and no prolongation of the thrombin time was observed [10, 11, 12]. The role of stabilized fibrin in the formation of intrapericardial fibrin deposits is marginal.

The administration of fibrinolytic agents into the pericardial space may potentially cause severe bleeding into the pericardial sac. Therefore, intrapericardial fibrinolytic treatment in patients with PP should be applied with caution, especially in patients with inflammatory chest disease, chest trauma or surgical therapy in recent anamnesis. One should also remember about contra-indications to the administration of a fibrinolytic agent (i.e., major haemorrhage or major trauma; coincidental stroke; major surgery in the previous 5 days; blood pressure >200/100 mmHg) [17].

According to ESC Guidelines 2004 and 2015 some patients require more invasive procedures like pericardiectomy. Pericardiectomy should be considered in patients with dense adhesions, loculated and thick purulent effusion, recurrence of tamponade, persistent infection, and progression to constriction [2, 3]. Surgical mortality up to 8% was reported for pericardiectomy combined with antibiotic treatment but the total mortality is even higher [2, 3].

## 6. Conclusions

The efficacy and safety of fibrinolytic drugs administered directly into the pericardial sac in PP require confirmation in randomized multicentre prospective clinical trials.

It seems however, that in the case of long-term, significant (more than 50 mL) purulent pericardial drainage, application of r-tPA directly to the pericardial space may be a breakthrough in the treatment of PP, without serious complications.

Safety of this procedure was confirmed on small group of treated patients and no influence on systemic fibrinolytic system and coagulation system was documented. In the longest follow-up, lasting in one patient for more than 7 years, no echocardiographic signs of CP were found.

The dosage of the drug and the method of its administration should be based on the previous experience resulting from the published papers.

Fibrinolytics appear to be highly effective in the prevention of pericardial constriction, but long-term data on a larger group of patients are needed.

## References

[1] Ferreira dos Santos L, Moreira D, Ribeiro P, Rodrigues B, Correia E, Nunes L (2013). Purulent pericarditis: a rare diagnosis. *Revista Portuguesa de Cardiologia*.

[2] Adler Y, Charron P, Imazio M, Badano L, Barón-Esquivias G, Bogaert J (2015). 2015 ESC Guidelines for the diagnosis and management of pericardial diseases: The Task Force for the Diagnosis and Management of Pericardial Diseases of the European Society of Cardiology (ESC)Endorsed by: The European Association for Cardio-Thoracic Surgery (EACTS). *European Heart Journal*.

[3] Maisch B, Seferović PM, Ristić AD, Erbel R, Rienmüller R, Adler Y (2004). Guidelines on the diagnosis and management of pericardial diseases executive summary; The Task force on the diagnosis and management of pericardial diseases of the European society of cardiology. *European Heart Journal*.

[4] Mann-Segal DD, Shanahan EA, Jones B, Ramasamy D (1996). Purulent pericarditis: rediscovery of an old remedy. *The Journal of Thoracic and Cardiovascular Surgery*.

[5] Sagristà-Sauleda J, Barrabés JA, Permanyer-Miralda G, Soler-Soler J (1993). Purulent pericarditis: review of a 20-year experience in a general hospital. *Journal of the American College of Cardiology*.

[6] Imazio M, Brucato A, Mayosi BM, Derosa FG, Lestuzzi C, Macor A (2010). Medical therapy of pericardial diseases: part I: idiopathic and infectious pericarditis. *Journal of Cardiovascular Medicine*.

[7] Rubin RH, Moellering RC (1975). Clinical, microbiologic and therapeutic aspects of purulent pericarditis. *The American Journal of Medicine*.

[8] Lazaros G, Vlachopoulos C, Lazarou E, Tsioufis K (2021). New Approaches to Management of Pericardial Effusions. *Current Cardiology Reports*.

[9] Cui H, Chen X, Cui C, Shou X, Liu X, Yao X (2005). Prevention of pericardial constriction by transcatheter intrapericardial fibrinolysis with urokinase. *Chinese Medical Sciences Journal*.

[10] Tomkowski WZ, Kuca P, Gralec R, Burakowski J, Caban P, Orlowski T (2003). Management of purulent pericarditis. *Monaldi Archives for Chest Disease*.

[11] Tomkowski WZ, Gralec R, Kuca P, Burakowski J, Orłowski T, Kurzyna M (2004). Effectiveness of intrapericardial administration of streptokinase in purulent pericarditis. *Herz*.

[12] Dybowska M, Kazanecka B, Kuca P, Burakowski J, Czajka C, Grzegorczyk F (2015). Intrapericardial fibrinolysis in purulent pericarditis-case report. *International Journal of Emergency Medicine*.

[13] Dybowska M, Szturmowicz M, Opoka L, Rudziński P, Tomkowski W (2020). Intrapericardial recombinant tissue plasminogen activator in purulent pericarditis- case series. *BMC Cardiovascular Disorders*.

[14] Augustin P, Desmard M, Mordant P, Lasocki S, Maury J, Heming N (2011). Clinical review: intrapericardial fibrinolysis in management of purulent pericarditis. *Critical Care*.

[15] Wiyeh AB, Ochodo EA, Wiysonge CS, Kakia A, Awotedu AA, Ristic A (2018). A systematic review of the efficacy and safety of intrapericardial fibrinolysis in patients with pericardial effusion. *International Journal of Cardiology*.

[16] Tsang TS, Califf RM, Stebbins AL, Lee KL, Cho S, Ross AM (1997). Incidence and impact on outcome of streptokinase allergy in the GUSTO-I trial. Global Utilization of Streptokinase and t-PA in Occluded Coronary Arteries. *The American Journal of Cardiology*.

[17] University of Cape Town IMPI 2 - A Trial of Intrapericardial Alteplase in Large Pericardial Effusion (IMPI-2). https://www.clinicaltrials.gov/ct2/show/NCT00265317.NLMidentifier:NCT02673879.

